# Machine-learning crystal size distribution for volcanic stratigraphy correlation

**DOI:** 10.1038/s41598-024-82847-0

**Published:** 2024-12-30

**Authors:** Martin Jutzeler, Rebecca J. Carey, Yasin Dagasan, Andrew McNeill, Ray A. F. Cas

**Affiliations:** 1 Centre for Ore Deposit and Earth Sciences, School of Natural Sciences, University of Tasmania, Hobart, Australia; 2 Datarock Pty Ltd, Melbourne, Australia; 3 Geological Survey Branch, Mineral Resources Tasmania, Rosny Park, TAS Australia; 4 School of Earth, Atmosphere and Environment, Monash University, Clayton, VIC Australia

**Keywords:** Mineralogy, Sedimentology, Volcanology, Economic geology, Stratigraphy, Geochemistry

## Abstract

Volcanic stratigraphy reconstruction is traditionally based on qualitative facies analysis complemented by geochemical analyses. Here we present a novel technique based on machine learning identification of crystal size distribution to quantitatively fingerprint lavas, shallow intrusions and coarse lava breccias. This technique, based on a simple photograph of a rock (or core) sample, is complementary to existing methods and allows another strategy to identify and compare volcanic rocks for stratigraphic correlation. Phenocryst size distributions display overall homogeneity within one volcanic body but may vary considerably between igneous bodies. Restricted to shallow intrusions and volcanic lavas, this concept allows for stratigraphic fingerprinting of volcanic rocks in poorly exposed, up to moderately altered, and/or complexly tectonized formations. We built an automated image analysis workflow using machine-learning for crystal segmentation, followed by statistical analysis of physical descriptors to compare and match the size distribution of feldspar phenocrysts. The workflow comprises three instance segmentation models for pre-processing the images, automated scale measurement and grain segmentation using Mask R-CNN. This avoids the laborious and time-consuming task of manual picking by image analysis, and allows for a rapid, unbiased and quantitative approach to determine crystal size distribution (CSD). Our volcanic architecture reconstruction of multiple dacite bodies in the mineralized Cambrian Mt Read Volcanics in Tasmania, Australia, is independently validated by bulk-rock chemical analyses of key samples. This volcanic stratigraphy method can be applied to a large variety of igneous rocks and is complementary to geochemical analyses and qualitative crystal content assessment.

**Supplementary Information:**

The online version contains supplementary material available at 10.1038/s41598-024-82847-0.

## Introduction

**Fig. 1:**
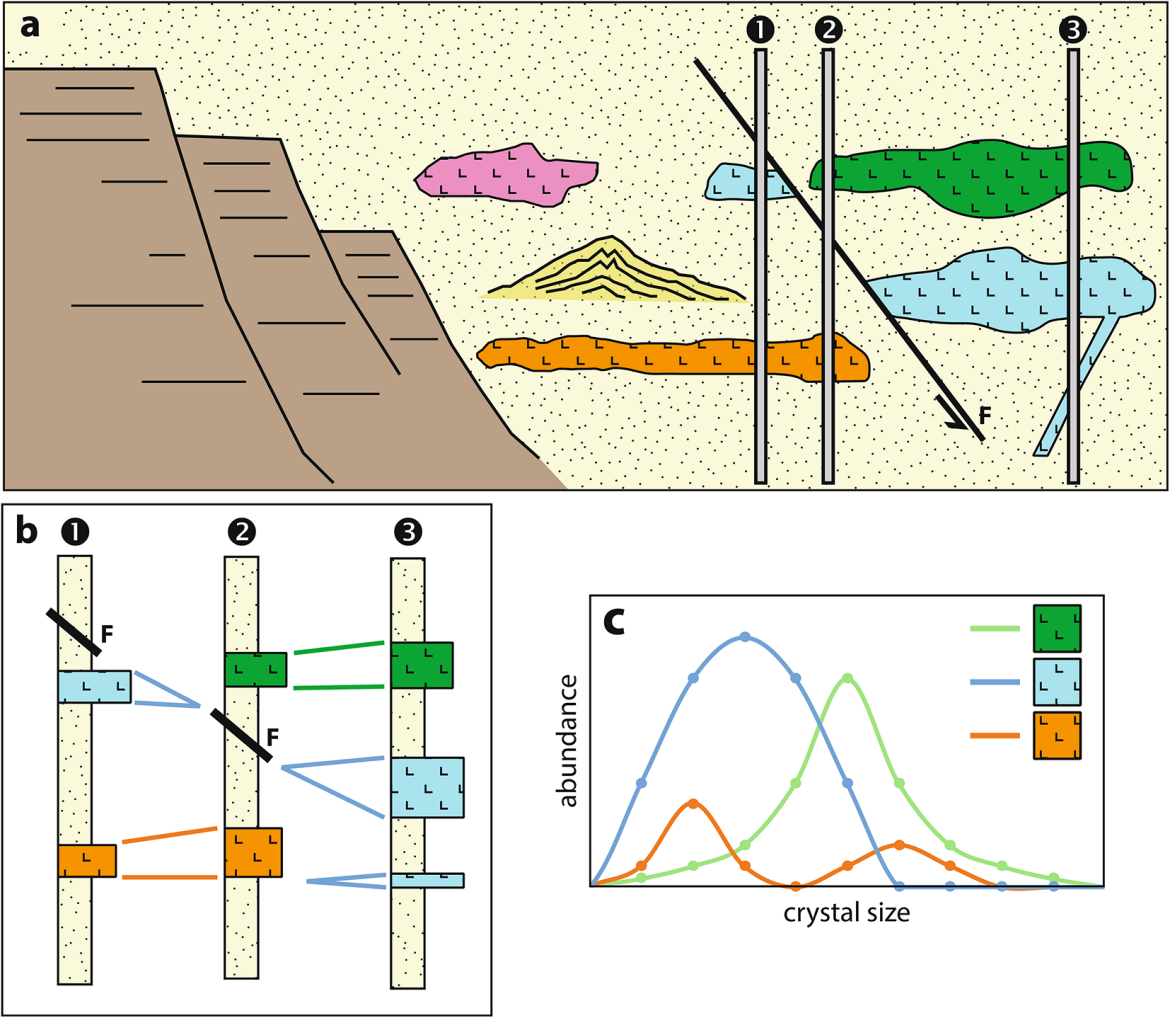
Idealized concepts of stratigraphic correlations to reconstruct volcanic architecture at the scale of a volcanic basin. (**a**) Cross section of a volcanic succession comprising coherent (lava and shallow intrusions) dacite bodies and volcaniclastic facies in a several km-wide basin, and intersected by three drillholes 1, 2 and 3. (**b**) Graphic logs of the three drillholes displayed in a fence diagram. The graphic logs can be built based on volcanic textures, geochemistry and/or physical properties. (**c**) The three coherent facies have distinct crystal size distributions, allowing for their stratigraphic reconstruction. The crystal size distribution method presented in this study provides a quantifiable method to support identification of matching volcanic facies and improving stratigraphic correlations. Fault, F.

Volcanic facies analysis is the classical approach for lithostratigraphic correlations and reconstruction of volcanic architecture at local to regional scale in volcanic provinces^1–8^. The method comprises description and interpretation of bedforms, contact relationships and volcanic (micro-)textures, and is commonly complemented by structural and geochemical data (Fig. 1). To identify the lithostratigraphic framework and to reconstruct volcanic architecture in Volcanic-Hosted Massive Sulphide (VHMS) and epithermal systems is critical for vectoring towards the mineralization^5–7,9,10^, and also used in geotechnical projects. The traditional descriptive approach in volcanic facies analysis has limitations due to the subjectivity of field observations and subsequent naming and comparison of facies based on their textures^2^. Geochemistry is the classic recourse to quantify lithological relationships^11^; however this technique becomes also limited when comparing rocks that are geochemically similar and/or are variably altered^9,12^.

Here we present a novel facet of lithostratigraphic reconstruction using semi-automated machine-learning segmentation of feldspar phenocrysts in porphyritic volcanic rocks. This method allows for the rapid, unbiased and quantitative analysis of crystal size distribution (CSD) based on routine digital photos of hand specimens and drill cores. Alkali and plagioclase feldspar phenocrysts are *the* ubiquitous minerals in a wide range of mafic to felsic volcanic rocks^2^, and therefore ideal for this method. CSD has traditionally been used in igneous petrology to interpret processes such as magma storage, ascent, and cooling^13–16^ but, at our knowledge, it has never been attempted for lithostratigraphic correlations until now.

Lavas and shallow sub-volcanic intrusions are relatively small volume (< 1 km^3^) and have an homogeneous size distribution of phenocrysts, in contrast to large volume, deep intrusions (plutons) that commonly have heterogeneous CSD due to multiple magmatic pulses, schlieren and/or cumulates^17^. Importantly, CSD varies from one volcanic/igneous body to another in terms of abundance and mean phenocryst size^13^. The CSD method for lithostratigraphic correlations presented here has a priori assumptions of a homogenous distribution of phenocrysts within the same volcanic body, but variability between volcanic bodies. Microlites that nucleate and grow in response to decompression, cooling and volatile loss during eruption are likely to vary across a given volcanic body and cannot be used for CSD-based lithostratigraphic correlations^13^. Phenocrysts can grow slightly during ascent^13^, however the crystal size enlargement is minimal and below our image resolution. Porphyritic clasts in volcanic breccias retain the CSD of their parent lithology, and therefore can also be used for CSD-based lithostratigraphic correlations.

Traditional CSD is calculated by manual hand contouring of individual crystal boundaries based on (a) density contrasts and/or chemical thresholds by Scanning Electron Microscopy (SEM)^13,18^; or (b) by density contrast using micro-tomography^19^. These techniques allow for precise CSD calculations of phenocrysts and microlites, however they are labor and time intensive, based on non-altered rocks, and the area of investigation is restricted to a few cm^2^ and thus arguably lack in statistical accuracy for phenocryst populations with individual crystal sizes greater than a few mm. Recent studies have used semi-automated CSD calculation based on SEM and micro-tomography images^19^; however the area of investigation is also limited in size, and recrystallization due to metamorphism and alteration bring complex issues for the identification of the original crystals. Complementary to 2D crystal identification, microscopy-based techniques require stereology calculations as an additional step to convert 2D populations into 3D statistical assemblages^20–23^.

Over the last decade, machine learning has become an essential tool for complex and repetitive recognition tasks to segment (i.e., delineate) specific objects from a background^24^. The method is particularly applicable to the geosciences including mineral exploration, where large numbers of samples are available, and textural identification is the basis of rock classifications. Deep-learning models allow for fast and automatic object recognition based on a series of teaching libraries prepared by a trained domain expert^25^. The general limitations of machine learning algorithms include rocks with unrecognized textures and/or alteration colors, or misidentification of objects; therefore manual quality control of the machine learning outputs by trained geoscientists is generally recommended^26,27^.

## Machine learning for computation of crystal size distribution

Volcanic facies analysis allows for lithostratigraphic correlations across basins (Fig. 1) and is critical for the reconstruction of volcanic architecture, and the identification of the main eruptive centers, coherent bodies and sedimentary depocenters present in a volcanic setting. Volcanic facies analysis provides qualitative and quantitative information that is fundamental to interpreting the nature and provenance of volcanic rocks, and to infer the 3D geometry of a volcanic sequence and its associated basins. This study focusses on feldspar phenocrysts, which are the most abundant type of crystals in volcanic rocks^2^, and therefore allow for a widespread use. The use of the size distribution of phenocrysts as fingerprint, allows for stratigraphic correlation of individual coherent volcanic bodies (lava flows and shallow intrusions) and their clastic associates across terranes.

**Fig. 2:**
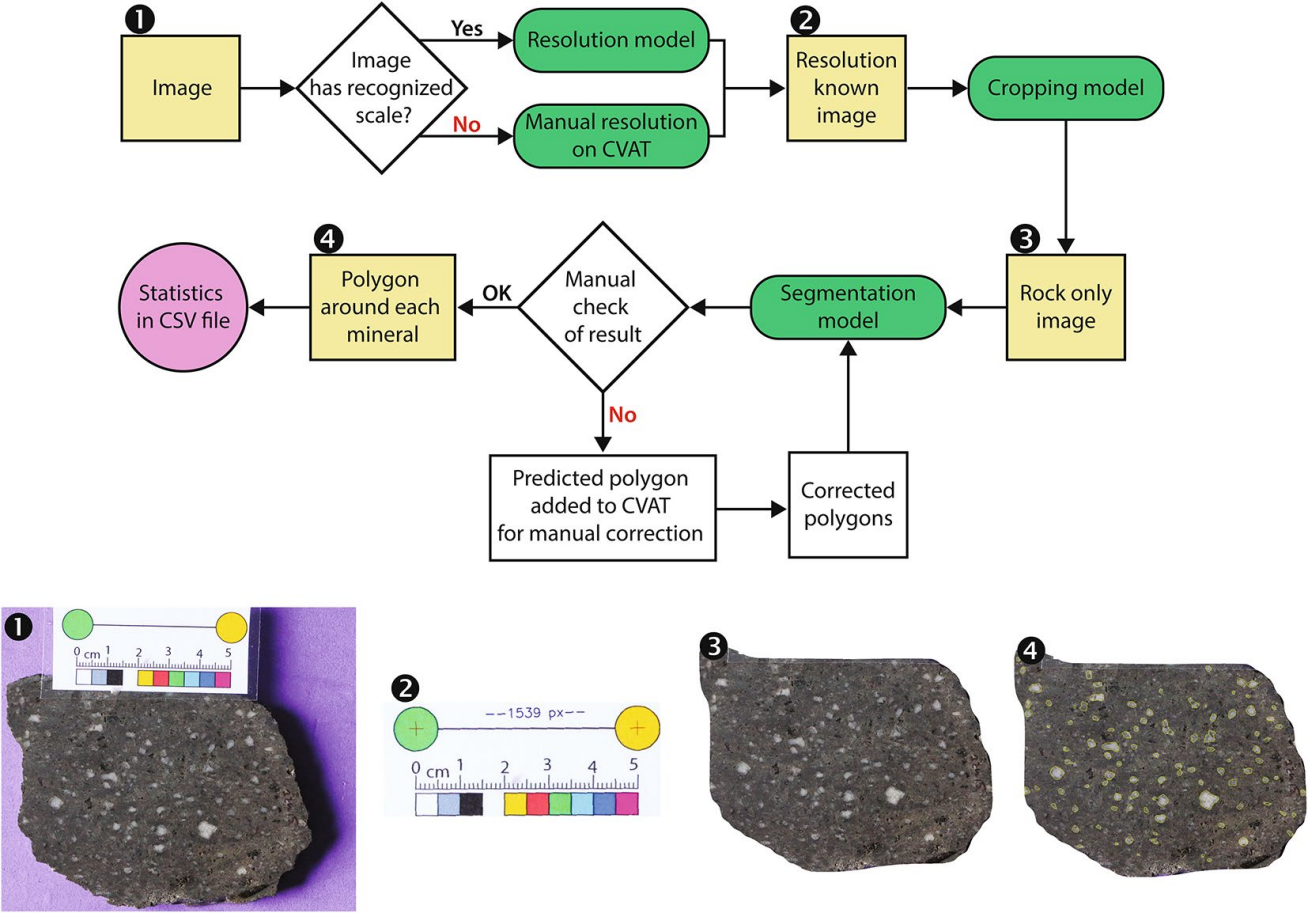
Visual workflow of the Machine Learning models (top) and output image for each step (bottom). Our image analysis workflow includes three machine learning models (green ovals): a resolution model based on a standardized scale adjacent to the rock sample; a cropping model that crops the area of interest from the background; and a segmentation model that identifies and contours feldspar phenocrysts. A manual check is embedded in the workflow, allowing a geoscientist to check and amend segmentation. A variety of crystal shape outputs are generated as a CSV file. The yellow outlines in image 4 are the segmented feldspar phenocrysts. CVAT is the Datarock online labelling platform (datarock.com.au).

We used machine learning algorithms to calculate the CSD of felspar populations in fresh and altered volcanic rocks in collaboration with Datarock Pty Ltd (datarock.com.au). Our machine learning strategy enables rapid and automated gathering of CSD data through repetitive tasks, producing outputs with limited user bias (Fig. 2). Our image acquisition workflow does not require extensive resources to ensure practicability, readiness, and reproducibility between users. Photos are taken on any wetted, flat hand sample with a labelled scale specifically designed for automated acquisition (known space in between two objects on the labelled scale). No further sample preparation is needed. Using a moderate image resolution, such as that of a modern mobile phone, we can identify crystals as small as 0.1 mm over an area of approximately 50–200 cm^2^, which translates to roughly 50 px/mm. This resolution captures most phenocrysts and therefore forms a statistically significant sample set. Images acquired continuously down core can also be used as sample set. Crystal sizes below the 0.1 mm threshold are discarded because their identification in altered rocks becomes subjective without microscopic analysis, are commonly recrystallized, and this grain size approaches thresholds in camera resolution.

**Fig. 3:**
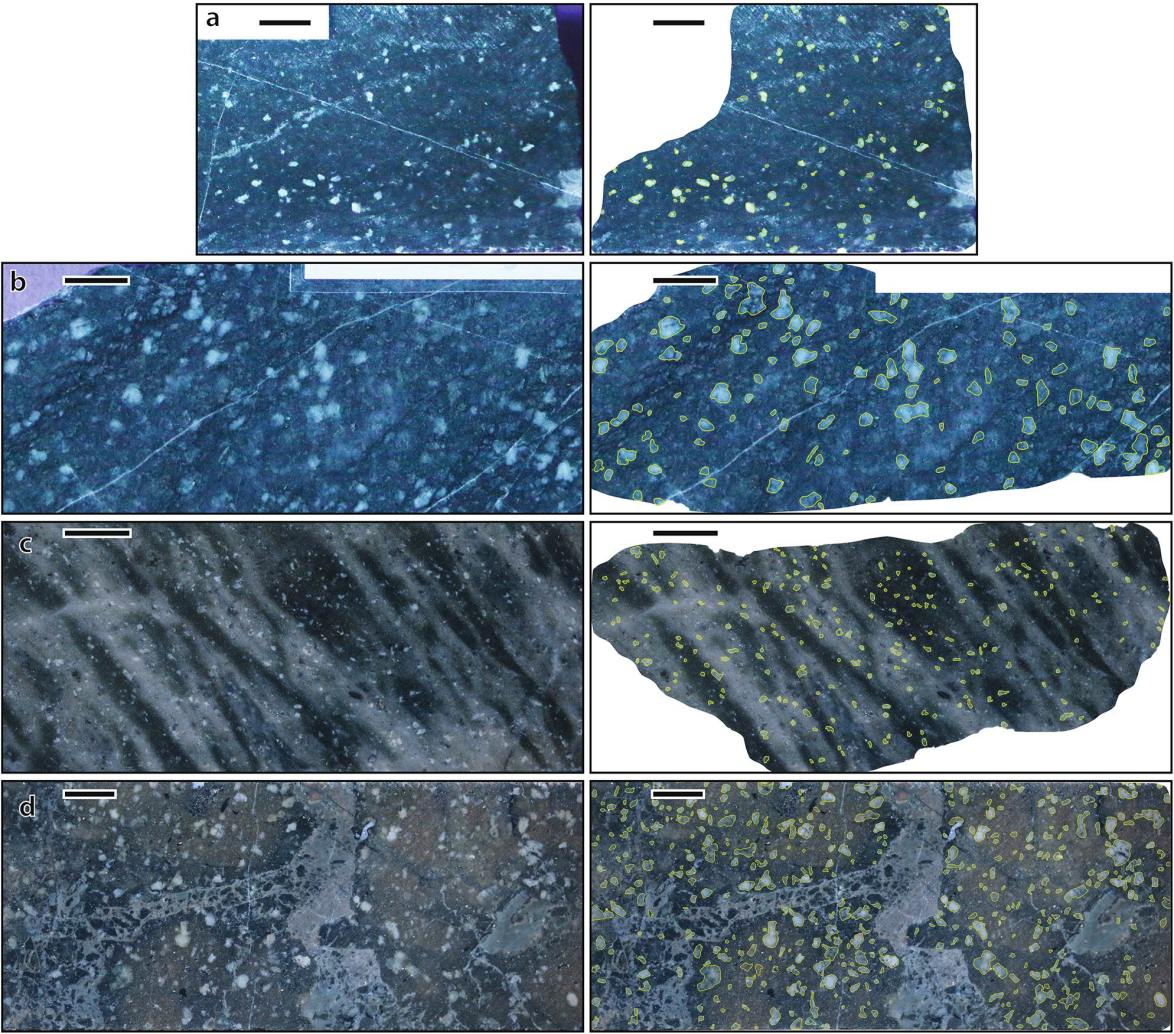
Crystal segmentation by machine learning on feldspar phyric rocks from the Mt Read Volcanics. Left, original photo, right, machine-learning segmentation. The yellow polygons contour feldspar phenocrysts surrounded by a recrystallized, metamorphic groundmass. Color and textural variations highlight the large spectrum of rock alteration that our machine learning technique was trained for, allowing for global applications (see Electronic Supplement for further examples). a, WSP10_183m brown population in the southern sector; b, YWS-1_382m orange population in the southern sector; c, MAC-40_581m teal population in the northern sector; d, BHD-8_212m, green population in the northern sector. Colors refer to Figs. 5, 6 and 7. Scale bars are 1 cm.

Cropping the rock from the background and applying size scaling are achieved using two deep learning models using Mask R-CNN^28,29^ (Fig. 2; Electronic Supplement). We employed the open-source Detectron2 library^30^ for model implementation and training, specifically using the Mask R-CNN architecture, which is well-suited for image segmentation tasks in geological images. Mask R-CNN has demonstrated robust performance in geological contexts, such as the automatic segmentation of joints on tunnel face images^31^ and rock quality designation analysis for rock cores^32^. This architecture was chosen for its versatility in detecting mineral boundaries under diverse conditions, as is commonly used in geological studies. Automation of these steps ensures a rapid workflow, limits false positives, and achieves high reproducibility for CSD comparisons (Fig. 3; Electronic Supplement).

Importantly, the size of the rock sample, the camera resolution and model are allowed to differ between samples, as our image analysis workflow is based on a scaled image and takes the area of investigation into account. A minimum of 40 crystals is recommended to obtain statistically significant results. Tests between the machine learning code and manual segmentation using Photoshop software clearly demonstrate that this new method is much faster and more reliable (Electronic Supplement).

A third deep-learning model, also using Mask R-CNN^28,29^, was developed to identify and segment feldspar crystals from groundmass, cement-filled vesicles (e.g., amygdales) and hydrothermal veins (Electronic Supplement). The model is based on our extensive library of variably altered mafic to felsic rocks emplaced in mineralized terrains, namely the Cambrian Mt Read Volcanics (Tasmania, Australia)^33^, the Ordovician Cowal Igneous Complex (NSW, Australia)^34^ and the Miocene Waihi mine (Hauraki goldfields, New Zealand)^35^(Electronic Supplement). The deep-learning model was trained with 130 images including 19,147 feldspar crystals which contour were individually delineated. The training was carried out on rocks that widely varied in alteration intensity, the color of their components and groundmasses (white, grey, black, yellow, green, orange and red), and crystal concentration and shapes (Fig. 3; Electronic Supplement). Such wide variations in textural properties were used to ensure the method is applicable to a wide range of porphyritic volcanic rocks. Feldspar phenocrysts never share the exact same hue or shape compared to other components of the same rock, allowing for their visual distinction. Further, feldspars typically maintain their size and shape in pervasive, weak to moderate mica, clay, feldspar and carbonate alteration. Therefore, our method can be successfully used with altered crystals, as long as their external shape is not modified by alteration. Hydrothermal alteration can be highly heterogenous and zoned, and where strong tends to destroy crystals entirely, thus creating easily identifiable zones where crystal populations are depleted; those zones should be avoided or masked during image acquisition.

We included a quality-control step for trained geoscientists to check the output of the automated segmentation task, allowing to manually remove or add feldspar selections if required. Our workflow allows for such control, increasing confidence in the machine learning automation and thus meaningful data acquisition. This important step is performed on the online Datarock labelling platform (www.datarock.com.au) and may require up to a few minutes for each sample (Electronic Supplement). Once finalized, 50 shape and length descriptors are calculated per crystal using the *imea* Python package^36^ and exported as a csv file. Area and aspect ratios are largely preferred to shape descriptors because complex crystal shapes are beyond the segmentation accuracy. This method can be used for most feldspar-phyric rocks, however crystal identification becomes increasingly difficult in highly phyric rocks where crystals are in contact with each other, and in strongly altered rocks where feldspars are unrecognizable. Other difficulties arise where the rock is intensely fractured or does not present a fresh surface.

## Lithostratigraphic reconstructions using CSD

**Fig. 4:**
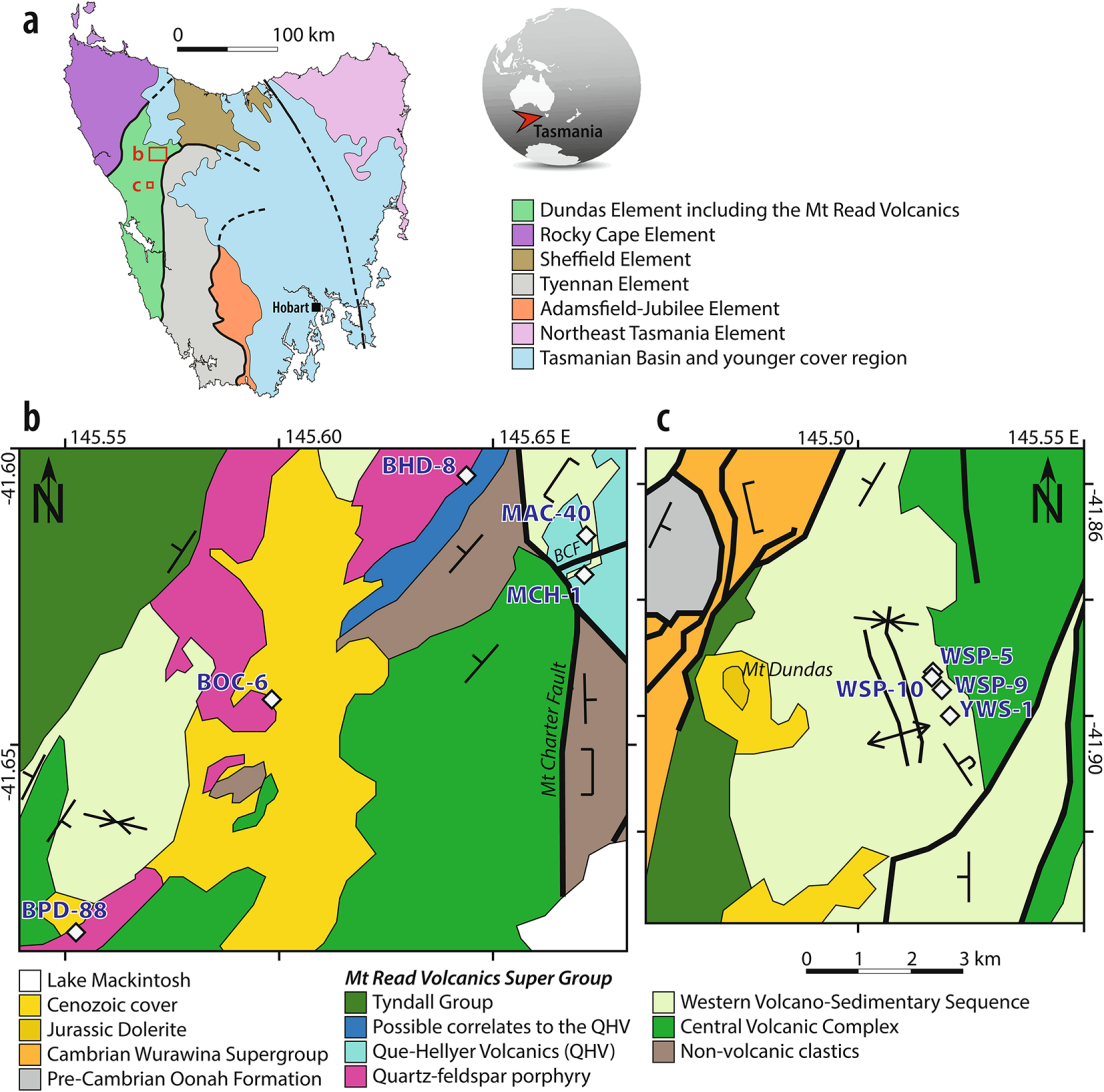
Location maps. (**a**) Simplified^45^ tectonic map of Tasmania, Australia. Red rectangles for northern (**b**) and southern (**c**) locations. (**b,c**) Two geological maps with the targeted drillholes in the Mt Read Volcanics (MRV) in western Tasmania, simplified^46,47^. (**b**) Northern locations; (**c**) southern locations. Drill core locations, white diamond; fault, thick lines; Barite Creek Fault, BCF. The original maps were digitized using Adobe Illustrator v. 27.3.1. https://www.adobe.com/au/products/illustrator.html.

The Mount Read Volcanics (MRV) is a middle Late Cambrian volcanic belt that records deep-water calc-alkaline volcanism and Zn-Pb-Cu-Ag-Au VHMS mineralisation within a wide extensional basin^5,9,33^. The MRV is dominated by coherent and clastic dacites and rhyolites (Fig. 3) with variable degrees of hydrothermal alteration^9^, which were subsequently subjected to deformation, greenschist facies regional metamorphism and areally restricted contact metamorphism from granitic bodies, during the Devonian Tabberabberan orogeny^33^. Lithostratigraphic reconstructions in the region of interest in the MRV have been achieved based on outcrops and drill cores^37,38^, however across-fault correlations between well-studied regions are ambiguous^39,40^. Clastic and coherent dacite-dominated sequences in the MRV are well exposed in nine drill cores over 34 km separated by a 23 km gap (Fig. 4). In the northern sector, several dacite bodies occur over five drillholes over a 15 km transect in a complexly faulted area^38,41^. In the southern sector, a group of dacite bodies exposed in four drill cores and partly outcropping have been proposed to be roughly contemporaneous, although not properly correlated stratigraphically^37,38^. For most dacite bodies, their emplacement as shallow intrusions or submarine lavas remains unverified due to poor contact preservation. We applied our method to 65 dacite samples across nine drill cores to assess the number of coherent bodies and their associated autobreccias, to reconstruct their lithostratigraphic relationships to one another, and in the context to the local and regional geology.

**Fig. 5:**
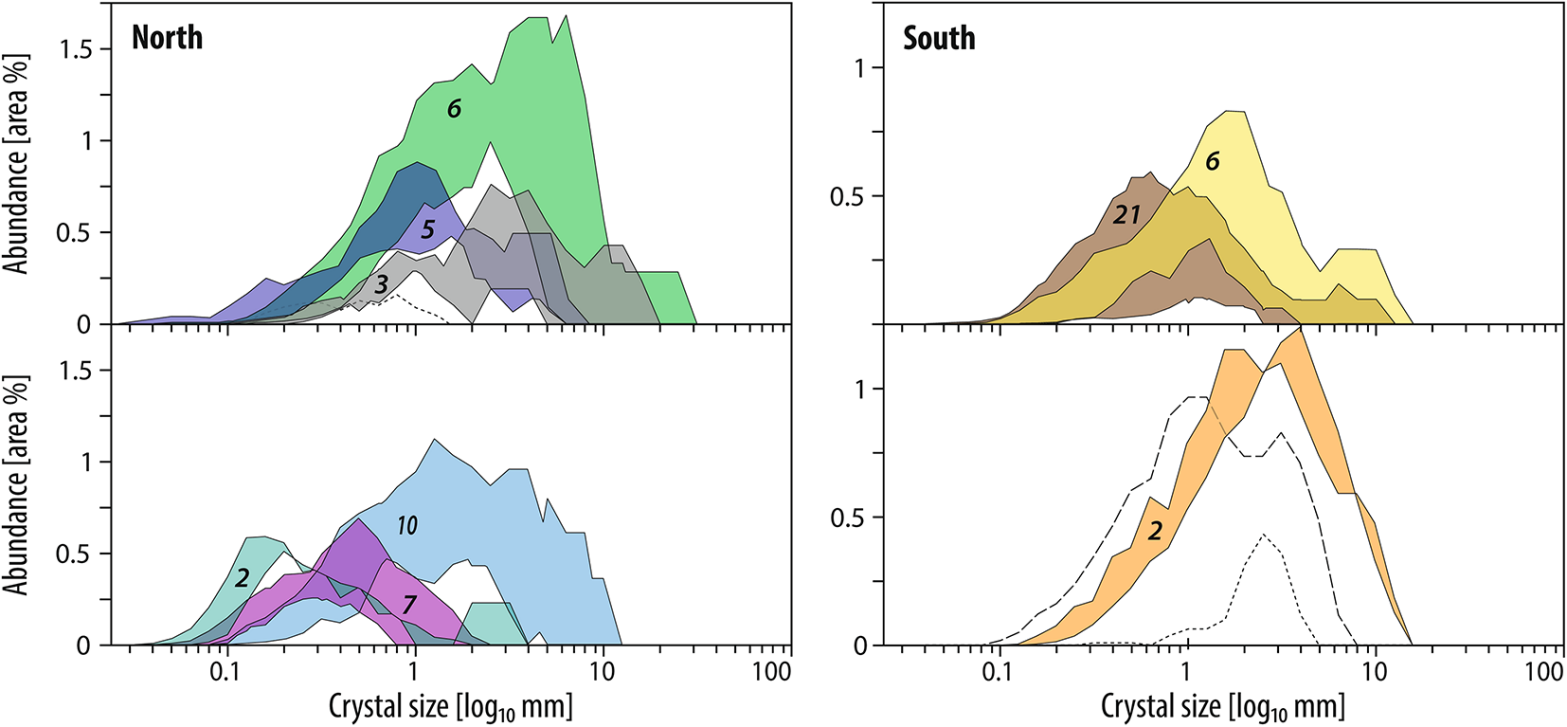
Grouped crystal size distribution (CSD) for feldspar phenocrysts obtained by our machine learning method. The histograms show feldspar distributions in the northern (left) and southern (right) sectors, Mt Read Volcanics, Tasmania (map on Fig. 4). Matching CSDs are grouped in colored envelopes; discrete units in dashed black lines. CSDs are displayed over two diagrams vertically as visual aid to identify group boundaries. Number of samples per group in italic. Symbols and color groups match those in Figs. 6 and 7.

**Fig. 6:**
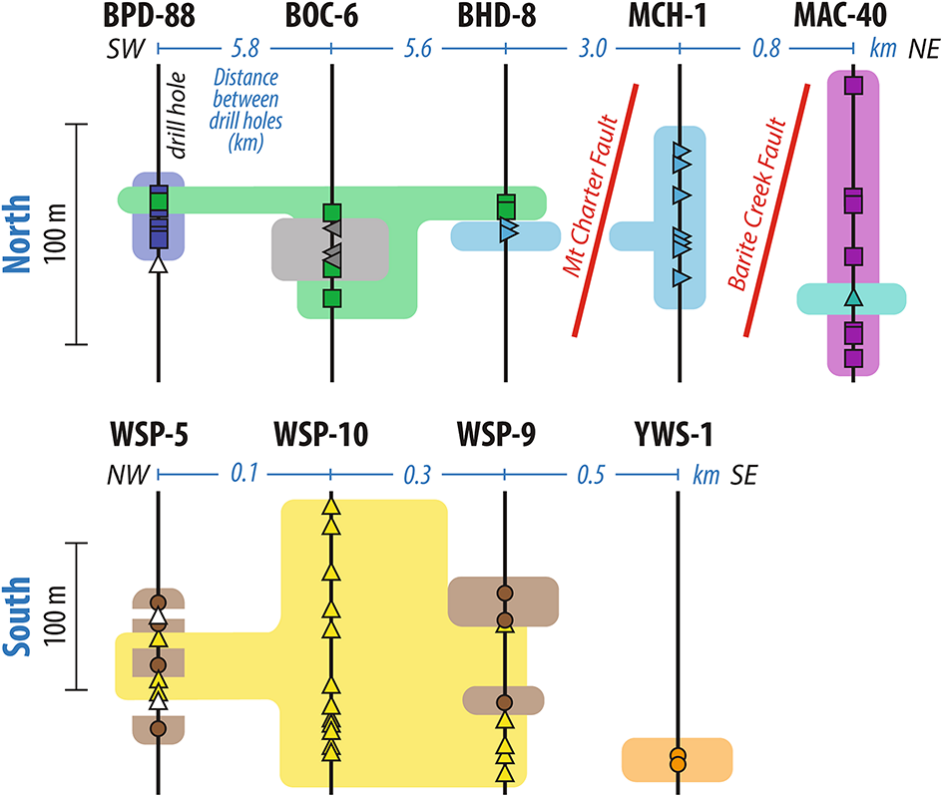
Stratigraphic reconstruction of 9 main dacite bodies (and three discrete units) in the Mt Read Volcanics, western Tasmania, based on crystal size distribution (CSD) identified in Fig. 5. Symbols and color groups match those in Figs. 5 and 7. White symbols for discrete units. Symbols on vertical black lines show the vertical location of samples in drill core; blue labels indicate distances (km) between drill cores.

Dacite bodies were picked out from one another through comparing the sample’s CSDs. The feldspar crystals range from 1 to 17% in concentration and 0.1–10 mm in size, although the minimum size corresponds to an attributed minimum threshold. Our analysis shows strong variations in CSD, allowing characterization of several dacite bodies (Fig. 5). The dacite samples were visually grouped based on the number, position and amplitude of the modes of their CSD, in addition to the general shape of their curves. Variations in crystal size distribution between samples, mostly pronounced through the amplitude (i.e., crystal content) did not allow automation of a statistical picking method. These variations in amplitudes are interpreted to reflect weak to moderate alteration that affect crystals regardless of their size, and thus still allows for successful CSD-based grouping. In the northern sector, six main dacite bodies were identified by CSD analysis of 33 samples, whereas three main dacite bodies were identified from 31 samples in the southern sector (Figs. 5 and 6). The CSD-reconstructed dacite bodies are meaningful geologically (e.g., extent, thickness and lateral continuity) and have associated dykes or sills that spread over hundreds of m to km laterally.

In the northern sector we identified six main dacite bodies with different CSD over five drillholes spread east-west over 15 km (Fig. 6). The regional Mt Charter Fault that was interpreted to have been active in the Cambrian^41^ runs southwest of MCH-1 and MAC-40 drillholes (Fig. 5). In this area, three distinct dacite bodies are crossed by MAC-40 and MCH-1 drillholes, despite being only 0.8 km apart laterally. MCH-1 and MAC-40 are separated by the local Barite Creek Fault (Fig. 4), also proposed to have been active in the Cambrian^41^. The dacite body of MCH-1 (pale blue) does not laterally correlate those in MAC-40 (purple and teal), supporting previous geological interpretations that this local fault crosses MAC-40 drillhole above the dacite body. The thin interval of dacite (teal) in MAC-40 has a different CSD to the identically similar dacite lithology above and below, and it remains unclear whether it is a sill or dyke, or a zone of intense alteration that modified the CSD within the same dacite body. Southwest of the Mt Charter Fault, three main dacitic bodies occur over more than 10 km distance in BPD-88, BOC-6 and BHD-8 drillholes. Interestingly, the dacite body from MCH-1 (pale blue) occurs as lower dacite body in BHD-8, despite these two drillholes being separated by the regional Mt Charter Fault, providing stratigraphic relationships between the various igneous bodies across this fault. Immediately above, a dacite body (green) is common to the three drillholes. Occurrence of a dacite body covering > 10 km is somewhat unexpected with the rare exception of large-volume caldera environment^42^. Further, its thin (< 6 m thick) expression in BPD-88 does not match the thickness of conventional highly viscous felsic lava morphologies^43,44^. We therefore consider the main dacite body (green) to lay over the < 6 km distance between BOC-6 and BHD-8 drillholes, whereas its expression in BPD-88 likely represents an associated sill intruding the main dacite body in BPD-88 (dark blue), instead of being an extrusive lava. The 20-m thick dacite body in BOC-6 (gray) and a discrete interval in BPD-88 are likely later dykes or sills intruding the main dacite body (green). The MAC-40 dacites were interpreted to be part of the Mixed-Sequence of the Que Hellyer Volcanics which hosts the polymetallic ore bodies^41^ (Fig. 4). All dacite bodies in the northern sector were previously interpreted by explorers to belong to the Mixed-Sequence as well^41^, hence making them highly prospective. Our phenocryst-based results however indicate that the dacite bodies southwest of the Barite Creek Fault are distinct from MAC-40 in terms of CSD (Fig. 6), and therefore may not be as prospective for mineral exploration as previously thought.

In the southern sector, four drillholes are spread across < 1 km (Fig. 6). A > 160-m thick dacite body (yellow) is present in three drillholes. It locally shows a brecciated top and is interpreted to be a crypto-dome^37^, and thins (to 30 m) at the WSP-5 drillhole. The complex contact relationship between the main dacite body and a secondary dacite body, common to both WSP-5 and WSP-9 drillholes (brown), may reflect magma mingling or pulses of two different dacitic magmas. The dacite body in YWS-1 drillhole (orange) occurs lower in the stratigraphy^37^ and our CSD study confirms it is a distinct body.

**Fig. 7:**
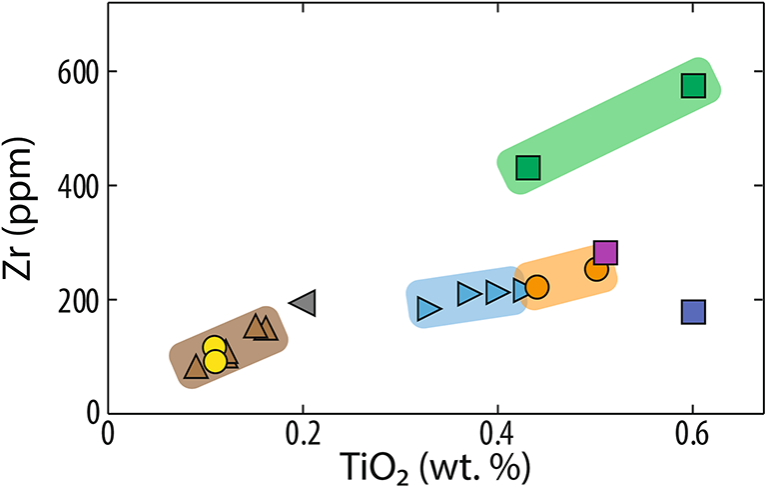
Comparison of crystal size distribution (CSD) analysis and bulk-rock geochemistry of immobile elements in dacites in the Mt Read Volcanics, western Tasmania. CSD outputs form compositional clusters, highlighting the excellent match in both methods. Symbols and color groups match those in Figs. 5 and 6.

Bulk-rock geochemistry on 21 samples independently validates our CSD-based interpretations (Fig. 7; Electronic suppl.). Compositional diagrams based on immobile elements (i.e., the least affected by alteration) show an excellent match with CSD-based stratigraphic reconstructions, with CSD-based units occurring in compositional clusters. This demonstrates that CSD can be a powerful tool for aiding stratigraphic reconstruction. These composition clusters have some range in compositions attesting to variable differentiation and/or crustal assimilation histories. Interestingly, the southern sector compositions are depleted in immobile elements, with the exception of the YWS-1 dacite that is chemically closer to the northern sector dacites. The tight cluster of chemical compositions in the dacite body that lies on both sides of the regional Mt Charter Fault (MCH-1 and BHD-8) confirms our CSD interpretation.

## Discussion

This study demonstrates that CSD analysis is a reliable approach that can be used in lithostratigraphic reconstruction in volcanic terrains that contain feldspar-phyric rocks (Fig. 7). Complementary and independent to traditional logging and geochemistry, CSD provides quantifiable datasets that allow for characterization of an intrinsic texture present in most volcanic rocks, and therefore of global significance. Its application to stratigraphic correlations provides an additional tool to improve volcanic architecture reconstructions, geological models and basin analysis to enhance strategies in mineral exploration, mining operations and other underground engineering projects. CSD allows for coherent bodies, such as lavas and shallow intrusions, to be distinguished from each other, but also to be linked to their associated coarse clastic facies. Geochemistry is extensively used for lithological fingerprinting^9,12^, however it produces compositional clusters and trends that commonly overlap, reducing its applicability to distinguish geologically distinct lithologies that are similar in composition. Further, alteration reduces the use of geochemistry to immobile elements, limiting the ability to differentiate between volcanic bodies. Likewise, two separate magmas may share a similar CSD, and locally strong alteration may decrease the visible abundance of phenocrysts, modifying the CSD. This may explain the similar geochemistry but variable CSD in the southern sector (yellow and brown dacite bodies), although these CSD variations may reflect local magma mingling or magmatic pulses in an endogenous volcanic dome, which is our preferred interpretation in this specific case. Combining CSD with geochemistry allows for an integrated interpretation that reduces false positives and selective biases in either of the techniques, producing a reliable method to correlate lithostratigraphic units in non-to strongly-altered feldspar-phyric volcanic rocks.

The limitations of CSD analysis reside in the degree of alteration of the rock, with the loss of phenocryst textures in highly altered rocks. Further, carbonate-filled vesicles form amygdales that can occasionally be difficult to distinguish from feldspar phenocrysts. Proposed future steps on this technique are the refining of the technique for detection of such amygdales, and to expand the machine learning technique to other phenocrysts, such as olivine, for applications in basaltic provinces for instance, and to distinguish clastic from coherent feldspar-phyric rocks based on crystal shape. Quality controls for accuracy and precision are routinely implemented by geoscientists in geochemical analyses, and we highly recommend implementing such controls on the machine learning outputs in CSD analyses.

## Electronic supplementary material

Below is the link to the electronic supplementary material.

Supplementary Material 1

Supplementary Material 2

## Data Availability

The datasets used and/or analyzed during the current study is available from the corresponding author on reasonable request.
