# Supplementary material for: Machine-learning crystal size distribution for volcanic stratigraphy correlation
Source: Sci Rep. 2024 Dec 30;14:31793. doi: 10.1038/s41598-024-82847-0 (PMC11685596; doi:10.1038/s41598-024-82847-0)
Supplement: Supplementary file 1 — Supplementary Material 1 [file 41598_2024_82847_MOESM1_ESM.pdf]

ELECTRONIC SUPPLEMENT

Machine-Learning Crystal Size Distribution for Volcanic Stratigraphy Correlation

Martin Jutzeler[1*], Rebecca J Carey[1], Yasin Dagasan[2], Andrew McNeil[3], Ray AF Cas[1,4]

1: Centre for Ore Deposit and Earth Sciences, School of Natural Sciences, University of Tasmania, Australia

2: Datarock Pty Ltd, Melbourne, Australia

3: Geological Survey Branch, Mineral Resources Tasmania, Rosny Park, TAS, Australia

4: School of Earth, Atmosphere and Environment, Monash University, Clayton, Victoria, Australia

Corresponding author*: Dr. Martin Jutzeler; *jutzeler@gmail.com*

LIST OF APPENDICES:

A - Learning library

B - Speed and accuracy of the method

C - Comparison of various image analysis methods

D - Reproducibility of the machine learning method

E - Sample list

F - Geochemical data

References

A – Learning library

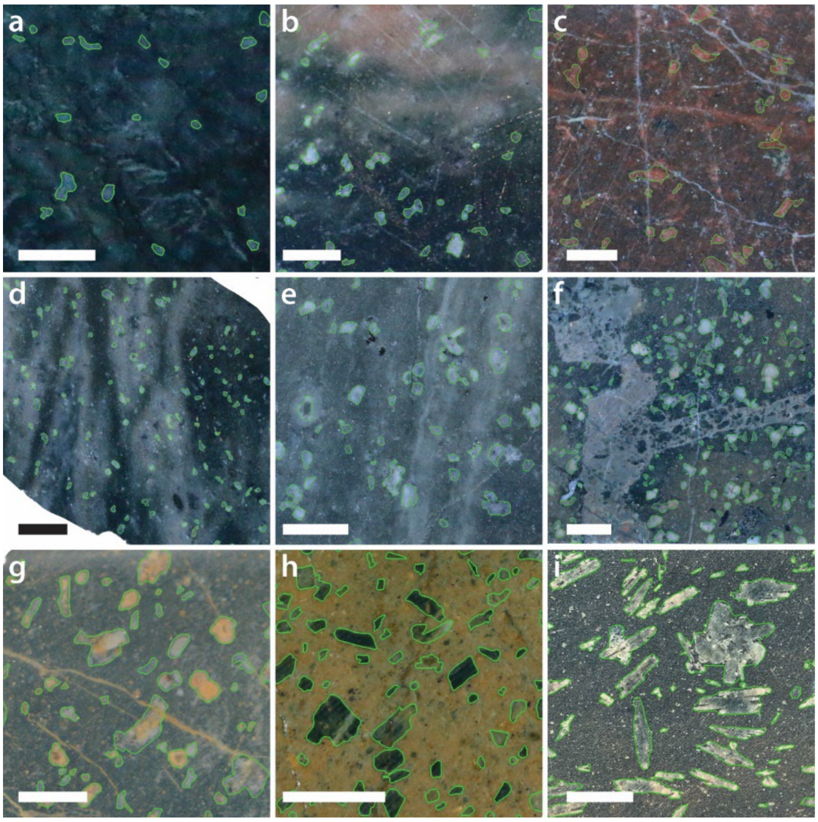

Fig. A1 *Example of training photos for feldspar phenocryst segmentation. The green polygons contour feldspar phenocrysts surrounded in a recrystallized, metamorphic groundmass. Color variations highlight the large spectrum of rock alteration that our machine learning technique was trained for, allowing for global applications. White scale bars are 1 cm. a-f, dacites from the mineralized Mt Read Volcanics, Tasmania, Australia; g-i, porphyritic rocks from the Cowal Igneous Complex, NSW, Australia, used in the training steps for their complexity in feldspar color, shape and size.*

B - Speed and accuracy of the method

We provide detailed information on the speed and accuracy of our machine learning models and compare it against two different 'manual' methods using the Adobe Photoshop software.

Our method comprises three machine learning models: cropping, scaling and segmentation. The machine learning model was trained on 19,347 feldspar crystals manually selected from 130 rock photos. This process took a considerable amount of time but resulted in a reliable machine learning model that identifies variably altered feldspar phenocrysts from multiple types of groundmasses (Fig. 2). Training the cropping model took 45 mins for 106 images, with predictions taking 1.1 second per image. The training for the scaling model took 30 minutes on 21 images, with predictions taking 4.06 second per image. The training for the mineral segmentation took 2.04 hours for 130 images, with predictions taking 4.82 second per image. Therefore, the automated part of the machine learning model takes a total of 9.98 second per image. All experiments were conducted using an NVIDIA RTX 2080 Ti GPU.

A trained geoscientist is required to check and improve the segmentation; this manual step less than 1 to 10 minutes per image, depending on the rock complexity, mineral abundance and efficiency of the segmentation model. The rock cropping segmentation has an Average Precision (AP) of 0.73 at intersection over Union (IoU)=0.50, the scale segmentation has an AP of 0.82 at IoU=0.50, and the mineral segmentation has an AP of 0.71 at IoU=0.50.

We carried out initial tests of feldspar segmentation using various tools in Photoshop and ImageJ software. The time required for manual cropping, scaling and segmentation carried out on varies enormously, depending on rock complexity, crystal size, and the ability of the Photoshop tools to differentiate between phenocryst and groundmass. Two main techniques can be used with Photoshop: 'manual picking' (using the Quick Selection tool), and the semi-automatic selection (using the Magic Wand tool); see Appendix C for additional information. Our experience shows that the Magic Wand tool only works where feldspar phenocrysts are in strong contrast with the groundmass, and that no complexity such as vesicles/amygdales and fractures are present, strongly limiting the use of this method. A total of 20-90 minutes is required per image and the analysis requires a geoscientist with good knowledge of Photoshop and ImageJ.

C – Comparison of various image analysis methods

The machine learning technique developed in this study allows for reproducible analysis of crystal size distribution (CSD) in volcanic rocks. Here we compare the outputs from three image analysis strategies to segment feldspar phenocrysts from their groundmass (Fig. C1). We strongly recommend using the *same* strategy for consistency throughout a study, with the machine learning method being the fastest (by far) and most reliable. The outputs of the three methods match relatively well, with matching main mode and that the three rocks can be visually distinguished from each other.

The outputs of the machine learning model show a coarse-grained peak that is not present in the Photoshop techniques (arrows in Fig. C1). Such secondary peaks are common where two crystals are merged during segmentation, which is allowed in our workflow to avoid additional biases that could arise from separating touching crystals. Importantly, such secondary peaks in the coarse-grained values have low statistical significance because they represent a few coarse crystals, and are thus not used to distinguish between CSD populations (see Appendix D).

The Photoshop techniques give satisfactory results for 'easy' rocks, where there is a high contrast between feldspar crystals and groundmass and no complexities such as vesicles/amygdales, fractures and/or veins. However, the Photoshop techniques (and in particular the semi-automatic method using the Magic Wand tool) become extremely unreliable in altered rocks. Furthermore, the segmentation rules will vary between users, increasing potential errors and biases.

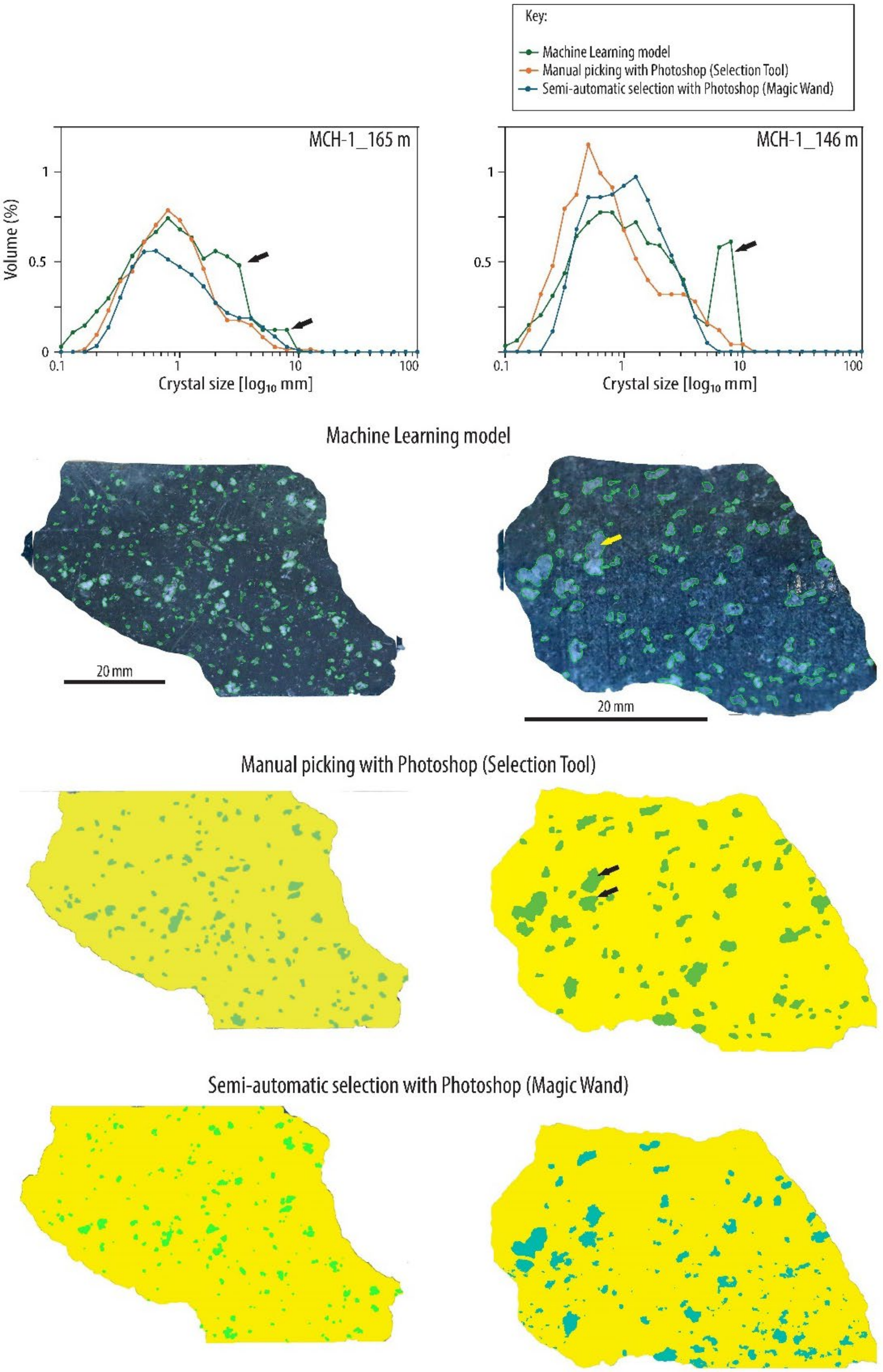

Fig. C1. *Comparison tests conducted between three segmentation methods. Top: CSD of the three methods. The Machine Learning method has excellent match with the two Photoshop methods, although positive peaks (arrows) in the coarse-grained feldspar crystals show that some crystal were merged compared to the Photoshop techniques. Such merging can be easily removed by a trained geoscientist in the post-segmentation check. The manual picking with Photoshop gives good results, however is time consuming an results will vary depending on the user. The semi-automatic selection tool in Photoshop gives similar results for easy rocks such as presented here but is unreliable for most rocks where the contrast between crystals and groundmass (and other textures) is low. Further, it is strongly dependent on the user and thus includes biases.*

It is interesting to compare the machine learning method and the semi-automatic selection with Photoshop (Magic Wand). Both methods show good match in CSD in an easy rock with good contrasts (Figs C1, C2), however extensive and time-consuming labor is required for the Photoshop method. The Magic Wand tool in Photoshop requires initial setup of a Photoshop file, manual filtering, setting up of contrasts and filters that vary for each photo, and manual clean-up followed by image recognition in ImageJ that include manual scaling, color threshold, and filtering for minimum grain size threshold. All filters and thresholds are dependent on the rock type, color and alteration, and therefore are difficult to reproduce between users. In many instances, the Photoshop Magic Wand tool is limited in its ability to select the correct fields, resulting in manual clean-up that can be more time consuming than to manually selecting each crystal with the Photoshop Quick Selection tool. The Magic Wand technique remains relatively slow (several minutes to tens of minutes to process in Photoshop and ImageJ). Further, we strongly recommend setting a standard minimum threshold for all rocks to avoid discrepancies in the distribution modes.

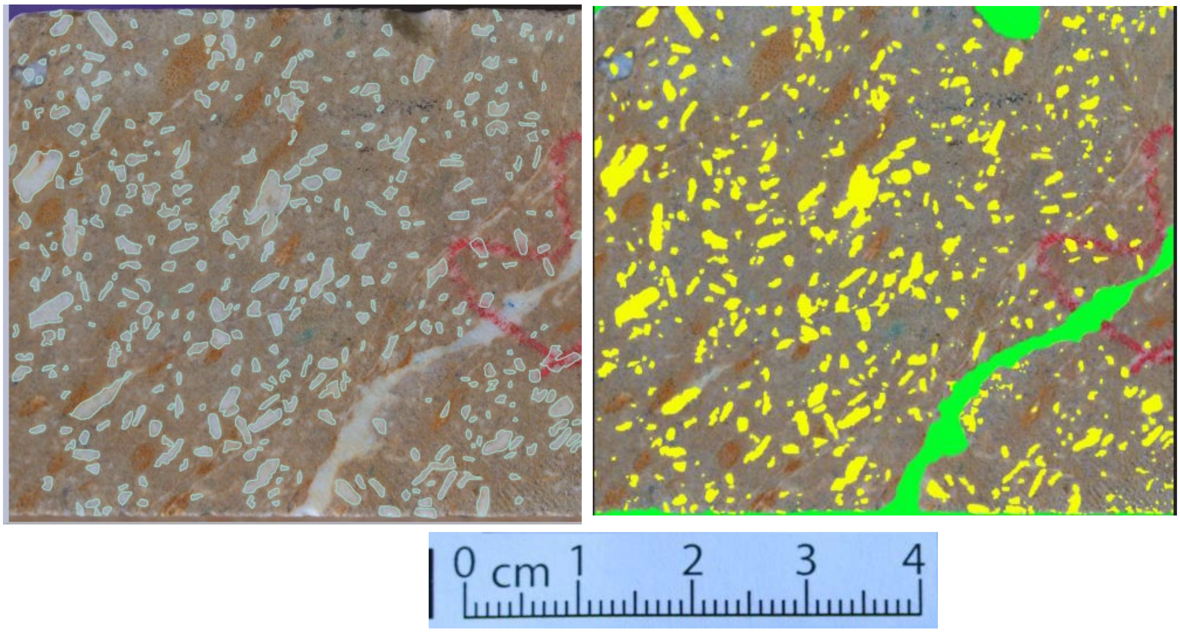

Fig. C2 *Test between Machine Learning (left) and semi-automatic selection with Photoshop (Magic Wand tool, right). The Machine Learning model allows for segmentation of most crystals, and quick clean up of wrongly attributed or missed feldspars during quality check. Segmented feldspar crystals outlines are in blue (left) and in yellow (right); quartz vein and background in green.*

D- Reproducibility of the machine learning method

The machine learning technique developed in this study allows for reproducible analysis of Crystal Size Distribution (CSD) in volcanic rocks. Reproducibility tests were carried out on unaltered, Miocene andesites from the Izu Peninsula[1,2], Japan. Three andesites were cut as 1-cm slices and 3 to 6 CSD analyses were carried out per rock (Fig. D1). The tests clearly demonstrate reproducibility in CSD analyses, and that each rock can be identified from each other. Scattering occurs in the coarse-grained field where phenocrysts occur in low numbers but have large volume. The presence of peaks in the coarse-grained regions serves as a secondary method for rock identification. Rock identification is based on the position and number of the main modes, which are well defined here.

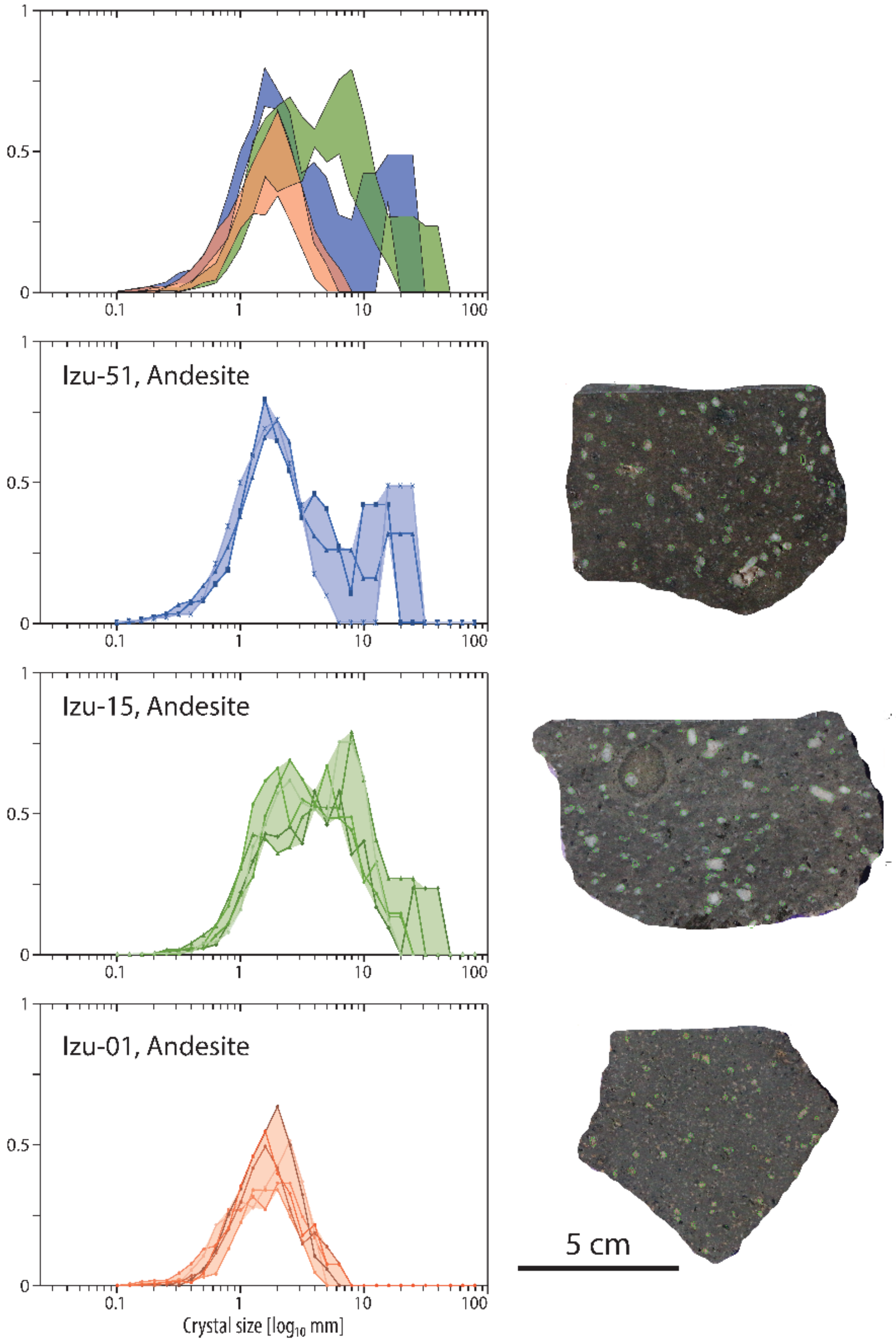

Fig. D1 *Reproducibility tests on three andesites. Lines show each CSD on slices in the same rock; colored field for maximum and minimum amplitude. The top graph compiles the three rocks.*

E – Sample List

South

ISOLATED	WSP-5	WSP-10	WSP-9	YWS1
	90			
	35			

YELLOW	WSP-5	WSP-10	WSP-9	YWS1
	22		53	
	36		35	
	64			
	107			

BROWN	WSP-5	WSP-10	WSP-9	YWS1
	46	23	56	
	74	37	107	
	83	68	120	
		92	137	
		106	144	
		143	156	
		157		
		165		
		167		
		173		
		183		
		188		

ORANGE	WSP-5	WSP-10	WSP-9	YWS1
				382
				388

North

ISOLATED	BPD-88	BOC-6	BHD-8	MCH-1	MAC-40
	101				

DARK BLUE	BPD-88	BOC-6	BHD-8	MCH-1	MAC-40
	72				
	78				
	87				
	90				
	91				

GREEN	BPD-88	BOC-6	BHD-8	MCH-1	MAC-40
	74	175	212		
		200	216		
		215			

GRAY	BPD-88	BOC-6	BHD-8	MCH-1	MAC-40
		183			
		194			
		199			

LIGHT BLUE	BPD-88	BOC-6	BHD-8	MCH-1	MAC-40
			222	125	
			226	132	
				146	
				165	
				168	
				169	
				171	
				183	

TEAL	BPD-88	BOC-6	BHD-8	MCH-1	MAC-40
					581
					583

PURPLE	BPD-88	BOC-6	BHD-8	MCH-1	MAC-40
					484
					535
					538
					561
					596
					597
					608

Table E1 *Sample list per sector and CSD group. Values correspond to meter below surface in diamond cores.*

F – Geochemical data

Whole rock XRF analysis of 21 dacites from drill cores in western Tasmania. The XRF methodology is detailed in the main worksheet. See attached spreadsheet (Table F1).

REFERENCES

1 Jutzeler, M., McPhie, J. & Allen, S. R. Submarine eruption-fed and resedimented pumice-rich facies: the Dogashima Formation (Izu Peninsula, Japan). *Bull. Volcanol.* **76,** 867 (2014).

2 Jutzeler, M., McPhie, J. & Allen, S. R. Explosive destruction of a Pliocene hot lava dome underwater: Dogashima (Japan). *J. Volcanol. Geotherm. Res.* **304,** 75-81 (2015).
